# Wing-dimorphism and population expansion of *Pterostichus melanarius* (Illiger, 1798) at small and large scales in central Alberta, Canada (Coleoptera, Carabidae, Pterostichini)

**DOI:** 10.3897/zookeys.147.2097

**Published:** 2011-11-16

**Authors:** Stephane Bourassa, John R. Spence, Dustin J. Hartley, Seung-Il Lee

**Affiliations:** 1 Department of Renewable Resources, 751 General Services Building, University of Alberta, Edmonton, AB, Canada T6G 2H1

**Keywords:** *Pterostichus melanarius*, wing-dimorphism

## Abstract

A study spanning ten years revealed changes in wing-morph ratios corroborating the hypothesis that the wing-dimorphic introduced carabid, *Pterostichus melanarius* Ill.,is spreading through flight, from the city of Edmonton, Canada and establishing populations in natural aspen forest of more rural areas 45-50 km to the East. Comparison of wing-morph ratios between *Pterostichus melanarius* and the native wing dimorphic species *Agonum retractum* LeConte suggests that the spatial variation in ratios for *Pterostichus melanarius* does not reflect underlying environmental variation, but instead the action of selective forces on this wing-dimorphic species. About ten years after its earliest detection in some rural sites the frequency of macropterous individuals in *Pterostichus melanarius* has decreased *c.* five-fold, but it is still above the level seen in European populations in which the two wing-morphs are thought to exist in equilibrium. *Pterostichus melanarius* is expanding its range in native aspen forest much faster than three other introduced species *Clivina fossor* L.), *Carabus granulatus* O.F. Müllerand *Clivina fossor* L also encountered in this study. The two *Carabus* species are flightless, but *Carabus fossor* can be dimorphic. Although these four non-native ground beetle species comprise >85% of the carabids collected at sites in urban Edmonton, activity-density of native carabids was similar across the urban-rural gradient, suggesting little direct impact of introduced species on the local abundance of native species. In a second study conducted at a smaller scale near George Lake, Alberta, macropterous individuals of *Pterostichus melanarius* have penetrated furthest and most rapidly into native aspen forest. Furthermore, the percentage of micropterous individuals has increased markedly in areas first colonized a decade previously. Overall, these studies support the idea that macropterous beetles in wing-d dimorphic species are important vanguards for early colonization of unexploited territory, but that flightless individuals replace the flying morph relatively rapidly once populations are established.

## Introduction

Many introduced species of Carabidae (Coleoptera) are thought to have arrived in North America from Europe in ballast of commercial ships (Lindroth 1957) or with nursery stock shipped to Canada from abroad before enforcement of effective quarantine regulations (Spence and Spence 1988). Once established, human-assisted dispersal among cities and towns likely played an important role in their spread and colonization of new areas (Niemelä and Spence 1991). These species have become a dominant part of the ground beetle fauna of Canadian cities and towns, and a subset are successfully invading other habitats, especially those disturbed by human activity (Niemelä et al. 1991; Hartley et al. 2007).

In some cases, this spread has been impressive and rapid. For example, the first Canadian record of *Pterostichus melanarius* Illiger is from 1926 in Nova Scotia (Lindroth 1961-1969), and a scant 50-60 years later the species had established populations from coast to coast (Spence and Spence 1988). The process of continental colonization was doubtlessly exacerbated by introduction of *Pterostichus melanarius* on the West coast, most likely through the port of Vancouver, and perhaps independently through ports on Vancouver Island (Spence and Spence 1988). First recorded in Edmonton by (Madge 1959), *Pterostichus melanarius* is now much more widespread in the province of Alberta and is among the commonest carabid species in cities, towns and many disturbed habitats in the southern half of the province (Niemelä and Spence 1991).

The ubiquitous distribution of *Pterostichus melanarius*, the size of its populations and its penetration of natural habitats prompts questions about the impact of this species on the structure of native carabid assemblages. Previous studies demonstrated significant impact of *Pterostichus melanarius* on native carabid assemblages in natural forests in Alberta (Niemelä and Spence 1991; 1994) and a laboratory study by Currie et al. (1996) suggested that intraguild competition and competition for food between *Pterostichus melanarius* and *Pterostichus adstrictus* Eschscholtz could potentially affect the population dynamics of these two species when they co-occur in nature. Establishment of *Pterostichus melanarius* in Canada is strongly associated with high human activity and its impacton native species seems greatest in anthropogenic habitats such as urban centers and agricultural land (Bourassa et al. 2008; Cárcamo et al. 1995; Cárcamo and Spence 1994; Hartley et al. 2007; Spence and Spence 1988).

The rapid and successful dispersal of wing-dimorphic species like *Pterostichus melanarius* is frequently explained in relation to selection promoting the dimorphism. For *Pterostichus melanarius*, brachyptery (possession of non-functional short hind wings, hereafter designated as ‘SW’) seems to be inherited as a simple Mendelian dominant gene (Aukema et al. 1996). In dimorphic species, this morph is thought to be favored through local competitive advantage, usually associated with more rapid reproduction (Spence and Spence 1988). The recessive gene at this locus codes for macroptery (functional long hind wings, or the ‘LW’ morph) (Aukema et al. 1996; Lindroth 1946). Hence, macroptery will be expressed only in individuals homozygous for the LW trait. Despite a study by Langor and Larson (1983) suggesting more complex gene interaction in the determination of wing length in other carabid species, the simpler Mendelian pattern seems to hold for *Pterostichus melanarius*. The LW morph is thought to persist by recolonizing populations extirpated by disturbance. In the case of introduced exotic species, this morph is important in spreading the species into uncolonized areas.

Although LW individuals are the exception in Europe (*c.* 2%) (den Boer and Van Dijk 1996), most North American populations of *Pterostichus melanarius* have a much larger portion of macropters. The dynamics of dispersal and colonization of new habitats by such wing-dimorphic species might be described as the arrival of “airborne” colonisers including LW females that had copulated with SW males before flight and therefore produce progeny of mixed wing length. As time passes, the SW gene is thought to become more prevalent in local populations, due to local competitive advantages of SW morphs. Therefore, for wing-dimorphic introduced and indigenous species, it may be hypothesised that a higher concentration of LW will prevail in recently colonized areas and that the age of a population will be inversely proportional to the frequency of LW individuals in that population.

In this paper, we consider these propositions with focus on populations of *Pterostichus melanarius* in central Alberta, Canada. In the first section, we consider variation in the proportion of LW individuals on an urban-rural gradient, starting in the city of Edmonton, the suspected point of initial colonization (Madge 1959), and extending 45-50 km into adjoining rural areas that have been successfully invaded by *Pterostichus melanarius*. In the second section, we study spatial and temporal changes in proportion of LW individuals at a smaller and finer scale, starting from the probable point of colonization along a road verge and extending into a native aspen forest. We build on earlier observations of Niemelä and Spence (1999) to examine temporal changes in distribution and abundance of the two wing-morphs.

## Materials and methods

### Landscape scale movement on an urban-rural gradient

Carabid populations were sampled during the summers of 1998, 1999 and 2007 along an urban-rural gradient using pitfall traps, as described by Hartley et al. (2007). Sites were selected in relatively continuous patches of aspen-dominated forest to fit one of the following three categories: (1) urban sites within the city limits of Edmonton, (2) suburban sites at the periphery of Edmonton and (3) rural sites 45-50 km from the city limits. Urban sites were along the North Saskatchewan River at a minimum distance of 2 km from each other. Suburban sites were within 20 km of the city centre (respectively, near St-Albert, in Sherwood Park, southwest of the city centre and south of the city centre). Rural sites were in Elk Island National Park and c. 15 km south of the Blackfoot Grazing reserve. Four sites were sampled in each category during 1998. In 1999, four sites were sampled in urban and suburban areas but only two rural sites were sampled, both in Elk Island National Park. In 2007 three sites were sampled in each category.

Ten pitfall traps were set per site in a transect with traps separated from each other by a minimum of 15 m to ensure independence of samples (Digweed et al. 1995). Each trap was 10 cm diameter, 15 cm in depth, was covered with a 12×12 square of plywood and contained approximately 3 cm of silicate-free ethylene glycol as a killing and preserving agent [see Spence and Niemelä (1994) for description of these traps]. Traps were open between 8 June and 24 August in 1998, 24 May and 23 August in 1999 and between 4 June and August 27 in 2007.

Trap contents were collected every 2 weeks and stored in 70% ethanol until processed. After sorting, all carabids were identified using Lindroth (1961-1969), and named following Bousquet and Larochelle (1993). A mix of native North American and introduced species [*Pterostichus melanarius*, *Carabus granulatus* L., *Carabus nemoralis* O.F. Müller and *Clivina fossor* (L.)] was captured. Wing length was recorded each year for *Pterostichus melanarius* and also for the native wing-dimorphic species, *Agonum retractum* LeConte, in 1998. We compared data for *Agonum retractum* and *Pterostichus melanarius* to test whether the patterns of wing-length ratio observed in *Pterostichus melanarius* might be reasonably interpreted as a response to some environmental gradient instead of in relation to the colonization and spread of a dimorphic species.

### Analysis of data from the urban-rural gradient

All carabids captured at each site along the urban-rural gradient during a particular season were pooled for analysis, i.e., data from each site represents the pooled catch from 10 traps. Percentage data (e.g., %LW) were arcsin transformed prior to analysis in order to meet assumptions of parametric analyses employed. The following statistical analysis were all performed using R version 2.12.2 (R Development Core Team 2010).

A two-way analysis of variance (ANOVA) was performed to evaluate differences in %LW individuals between *Pterostichus melanarius* and *Agonum retractum*. Tukey’s HSD post-hoc test was used to evaluate differences revealed by ANOVA. To investigate if %LW varied among sampling years in *Pterostichus melanarius*, 2- way ANOVA was conducted, using year and position on the gradient (urban, suburban and rural) as model factors. A Tukey-Kramer post hoc test for unbalanced designs was used to identify significant groups in the data. Another two-way ANOVA was performed on the 1999 data (the only year in which sex was recorded) for *Pterostichus melanarius* to test for significant differences in %LW between sexes along the urban-rural gradient.

In order to assess the potential impact of urbanization on the native carabid assemblage, ANOVA was performed to compare whole-season standardized activity density of all native species among the three positions on the gradient. For this analysis, activity density was standardized to the lowest number of undisturbed trapping days recorded at any site.

### Local scale movement at George lake

Movement of *Pterostichus melanarius* has been monitored using a pitfall grid in aspen forest near George Lake, Alberta (52.53°N, 112.10°W) since 1991. The study area and the general approach to trapping has been described in detail by (Niemelä and Spence 1999). The forest area studied is bordered on the south by a paved highway (Alberta Route 651). The highway has a large verge with grassy vegetation, which is the likely source of the original *Pterostichus melanarius* forest colonists, and on the west by a gravel farm access road that is bordered by tall grass.

In this paper, we present new data from this effort, as collected in 2007 and 2008.Niemelä and Spence (1999) describe the trapping designs for the year 1991, 1992 and 1997. In 2007, each transect included 5 traps spaced at 50 m intervals, starting 50m East of the gravel road and extending into the forest. The southernmost transect (T1) was in the verge adjacent to the highway and the second (T2) was 5 m inside the forest edge leaving only a distance of 10 m between T1 and T2. The following transects (T3-11) were spaced at 50m intervals, moving northward from T2 to a final transect at 450m. In 2008, each transect comprised 4 pitfall traps starting 10 m into the forest on the east side of the gravel road and extending east into the forest. In 2008, transects were as in 2007 but additional transects were added spaced at intervals of 100m to extend 950m northward in the forest.

Because our intent was to focus strictly on the population of *Pterostichus melanarius*, specimens were collected from the traps biweekly during the periods of 6 Jul to 1 Aug in 2007 and 14 Aug to 11 Sept in 2008. Although a few individuals of this species overwinter as adults and are active in the spring, the great majority of the annual adult activity is from overwintered larvae which emerge as teneral adults that are active and breed in Alberta during late summer. All carabids collected were identified to species throughout this study, except in 2007 where only *Pterostichus melanarius* was identified to species and all other carabids were recorded as “other carabids”. Wing length for all individuals of *Pterostichus melanarius* was recorded as LW or SW.

### Analysis of George lake pitfall trap grid

To compare the earlier data of from the 1990s to the data collected in 2007 and 2008, catches of the two later collections were standardized as described in Niemelä and Spence (1999). Because the trapping periods in 2007 and 2008 were shorter (targeted to capture *Pterostichus melanarius*) activity densities of *Pterostichus melanarius* were calculated by adding an extra 90 days to the divisor to arrive at a figure for catch/day. Additionally, in order to clarify broad temporal patterns, data from 1997 were compared with data averaged, respectively, for 1991 and 1992, and for 2007 and 2008. Pitfall trap samples from each transect were pooled to give a single value for %LW or activity density at each distance from T1.

The significance of temporal changes in %SW of *Pterostichus melanarius* was tested using a comparison of two proportions (Zar 1999) using data from 1991 – 1992 and 2007 – 2008. We compared the %SW captured for two sets of traps, those <100 m north of the highway and those >100m north.

## Results

### Landscape scale movement of *Pterostichus melanarius*

**Patterns of Wing-Dimorphism in *Pterostichus melanarius* and *Agonum retractum*.** Two-way ANOVA showed a significant interaction between ‘Gradient’ and ‘Species’ (P < 0.001) for transformed data about %LW in these two species. The %LW individuals was statistically higher for *Pterostichus melanarius* in rural sites than in either urban or suburban sites (Tukeys HSD, P<0.01) (Figure 1). Differences between urban and suburban populations were not as consistent (P = 0.070), although the average data certainly suggest existence of a gradient. There was no significant difference in the %LW for *Agonum retractum* across the gradient, suggesting that the clear pattern seen in *Pterostichus melanarius* did not result from underlying environmental variables that generally affect flight ability in local carabid populations. Also, we note that the %LW for *Pterostichus melanarius* was significantly higher than for *Agonum retractum*, but these means differed significantly only in rural sites (P < 0.001).

**Figure:**
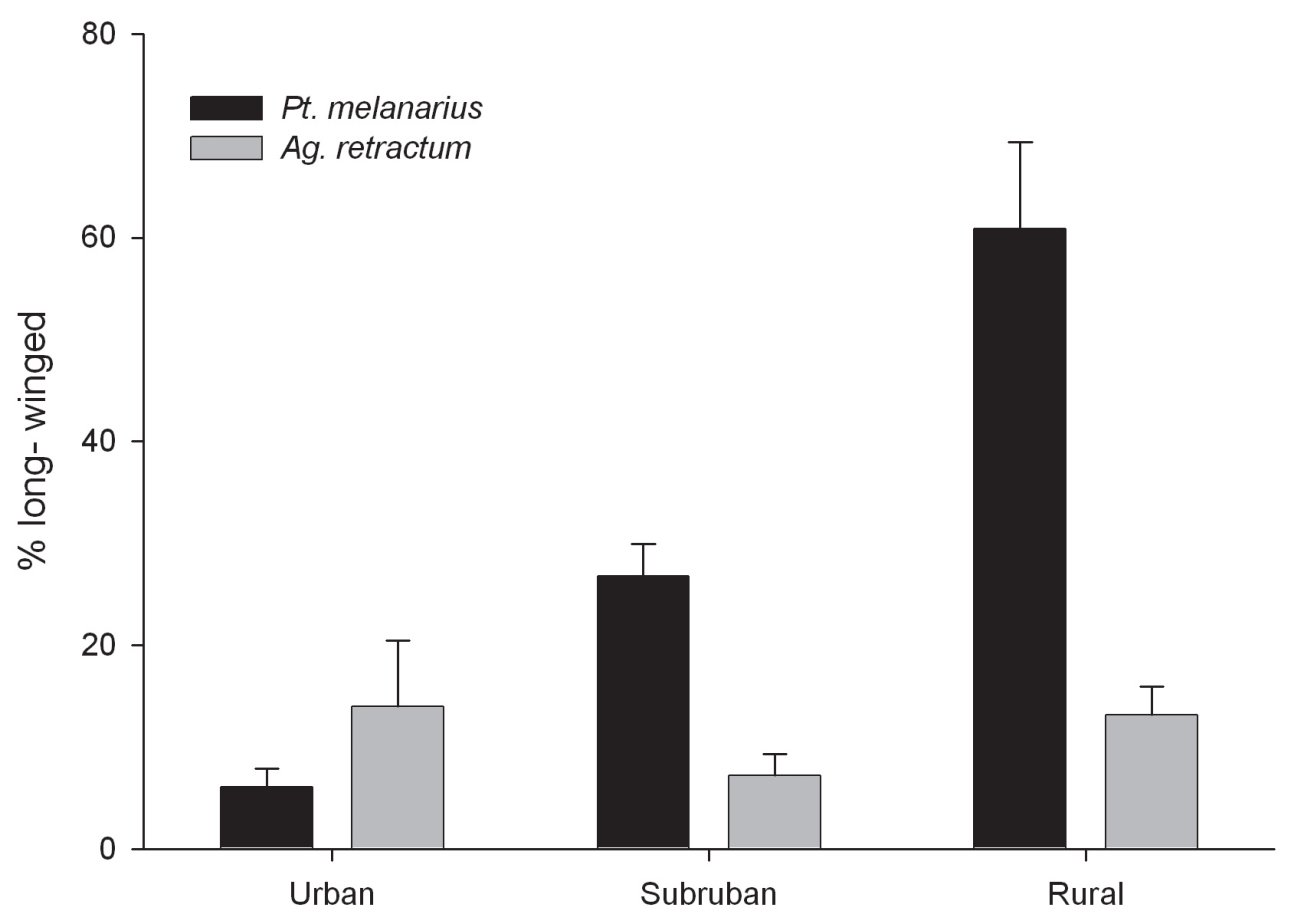
**Figure 1.** Percent LW individuals found in *Pterostichus melanarius* (a non-native species) and *Agonum retractum* (a native North American carabid) along an urban-rural gradient. Each bar represents the mean of 4 sites ± 1 S.E.

**Spatio-temporal changes in pattern of wing-dimorphism in *Pterostichus melanarius*.** There was significant interaction between the model factors ‘Year’ and ‘Gradient’ (P < 0.01) on %LW in *Pterostichus melanarius* between 1998 and 2007. A Tukey-Kramer multiple comparison test for unbalanced design confirmed that %LW was significantly lower in 2007 than in 1998 and 1999 in the rural sites (Figure 2). Thus, during this period, the %LW in *Pterostichus melanarius* decreased in rural sites but remained rather constant in both rural and suburban areas.

**Figure:**
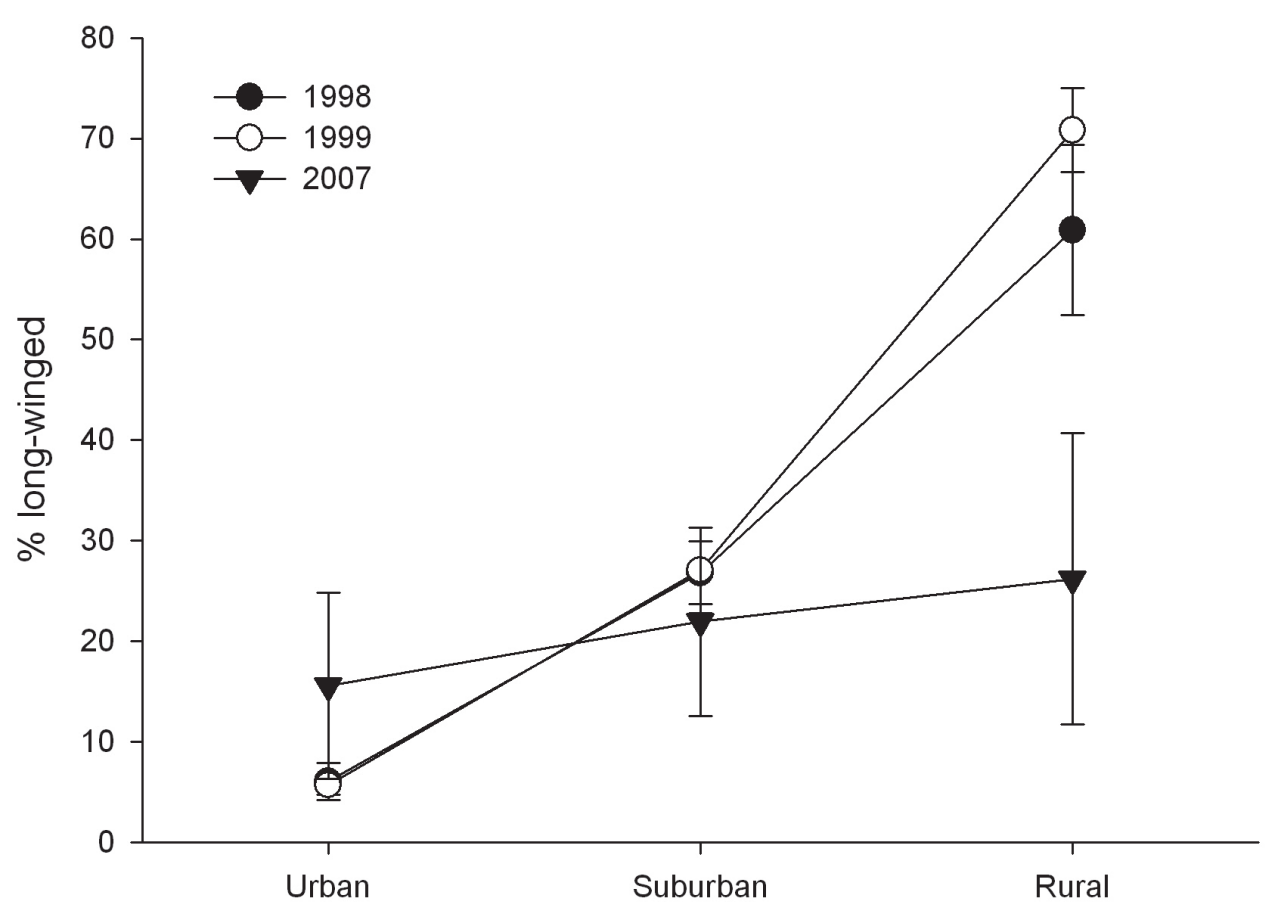
**Figure 2.** Comparison of %LW in *Pterostichus melanarius* along an urban-rural gradient over the years 1998, 1999 and 2007. Error bars are ± 1 S.E of the mean.

**Sex-associated differences in %LW in *Pterostichus melanarius***. There was a significant effect of ‘Gradient’ (P < 0.001) on the overall %LW in *Pterostichus melanarius* but neither ‘Sex’ nor the (‘Sex’ × ‘Gradient’) interaction was significant. Thus, given the small numbers of *Pterostichus melanarius* caught in the rural sites in 2007 (2 males and 3 females), we could not detect any significant differences in %LW between males and females.

**Distribution of introduced and native species over the gradient.** Figure 3 shows a clear pattern of decreasing proportion of non-native of species with increasing distance from the urban center. We detected the presence of only *Pterostichus melanarius*, among non-native species, in the rural sites in two of the three years sampled. At the urban sites, however, 85.3% of the carabid fauna was attributable to the four introduced species. Populations of all four were detected in the suburban zone where they constituted 49.1% of the carabid fauna. Thus, among introduced carabids in central Alberta, the wing-dimorphic species, *Pterostichus melanarius,* is the superior colonizer.

**Figure:**
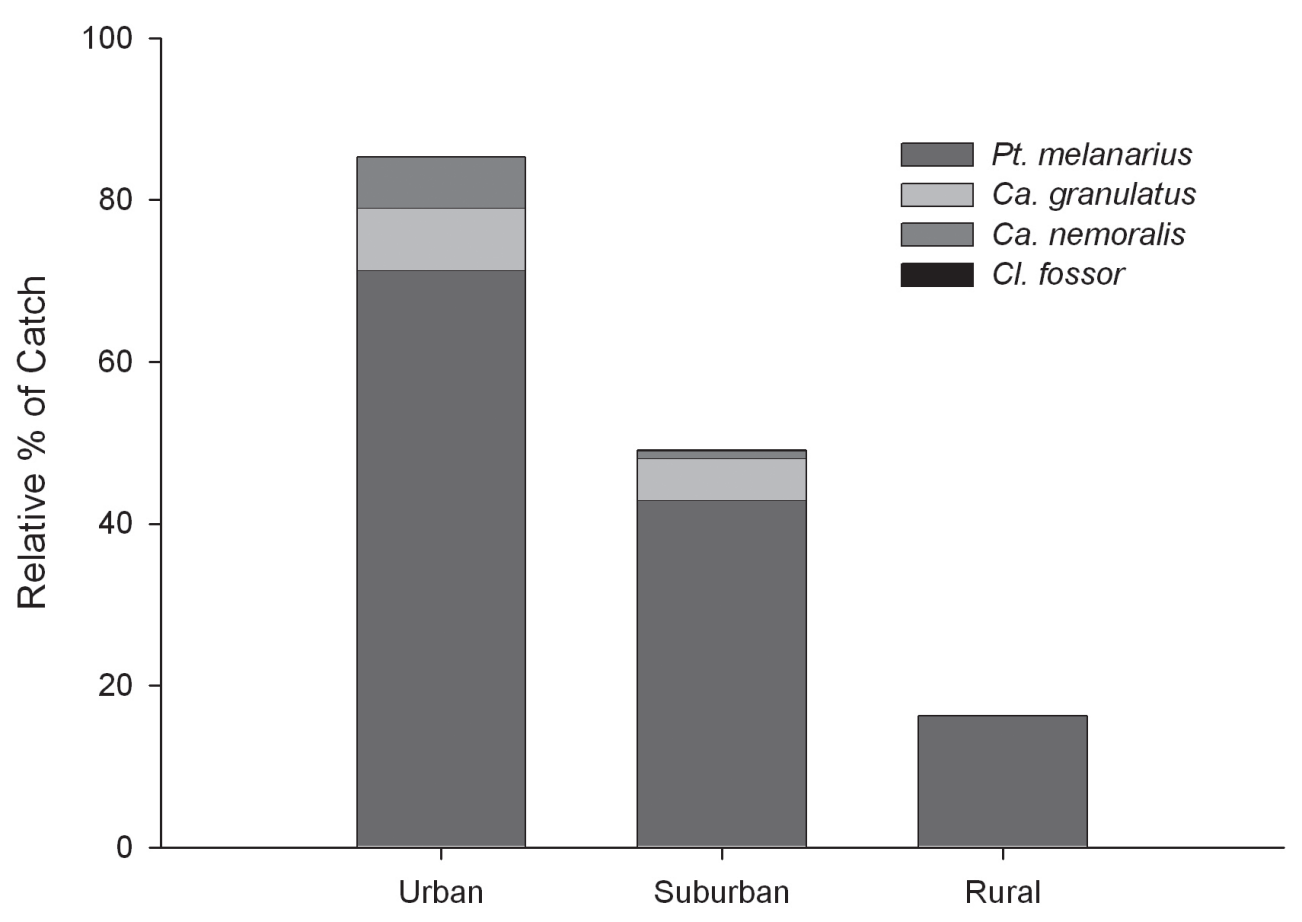
**Figure 3.** Representation of four introduced carabid species in the carabid fauna along the urban-rural gradient (1998-99).

Despite the numerical dominance of introduced species at urban sites there is little evidence for overall negative impact on the native fauna. There was no change in overall activity density of native species along the urban-rural gradient (p > 0.05) (Figure 4), despite the huge abundance of introduced species at the urban sites.

**Figure:**
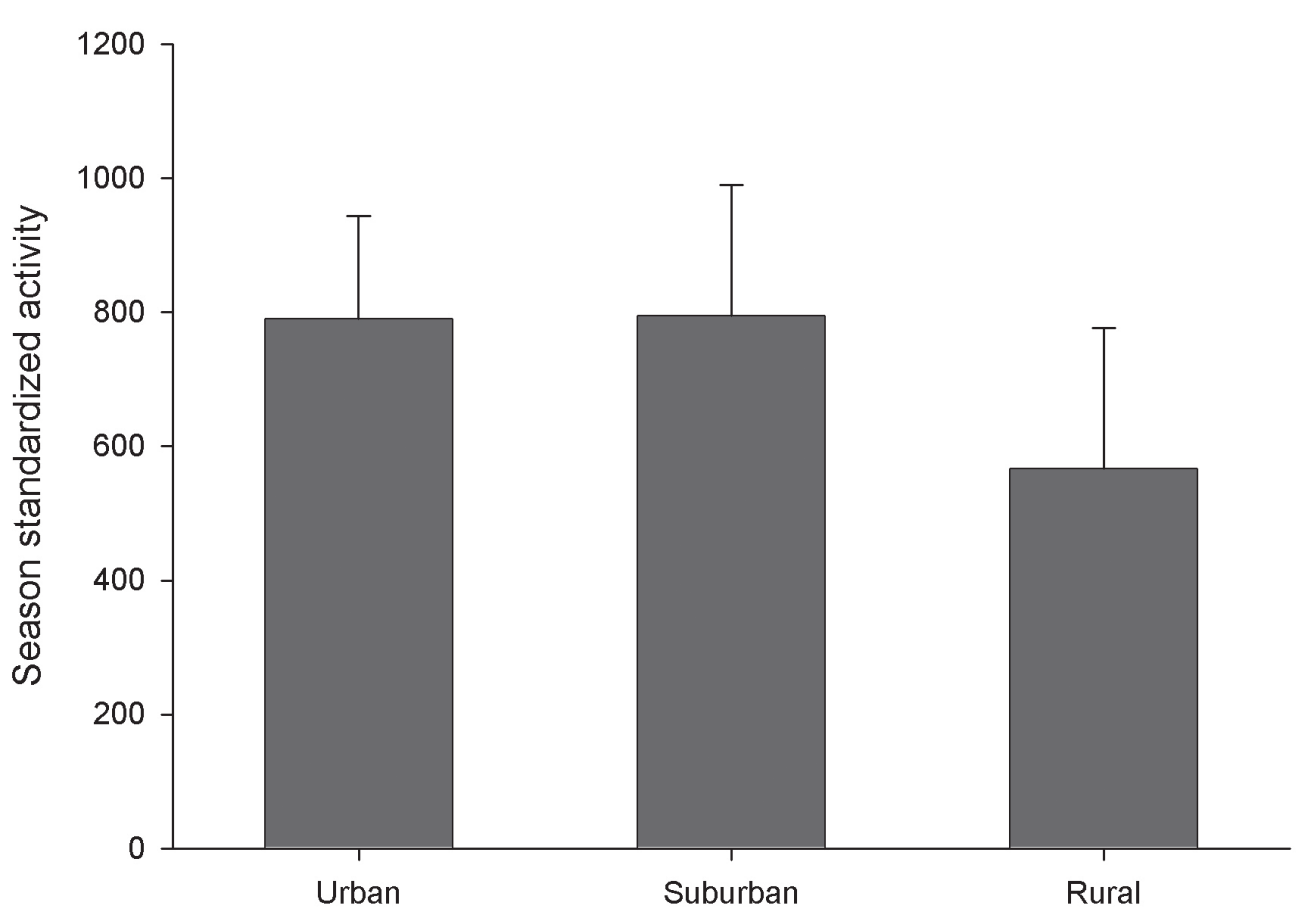
**Figure 4.** Mean activity density of all native carabid species along the urban-rural gradient (1998- 1999). Activity density is expressed as standardized whole-season catch (see text for details). Means exclude 2 rural sites that were not sampled in 1999 and are therefore an average of 2 sites. There were no statistically significant differences among activity in these three zones (ANOVA, p > 0.05). Error bars show standard error.

Small scale movement of Pterostichus melanarius

A total of 236 adult *Pterostichus melanarius* were collected in the George Lake pitfall grids, 134 in 2007 and 102 in 2008. In contrast to the earlier years (1991-92 and 1997), the vast majority of *Pterostichus melanarius* individuals were collected in the 5 m forest edge (46.6%) while only 11.4% of the individuals were collected in the grassy road verge (Figure 5a). A total of 10.5% of the individuals were captured 50 m into the forest, leaving only 31.5% of the catch to be distributed over trap sites ≥ 100 m into the forest interior.

A total of 21 carabid species was collected at George Lake in 2008, with the highest species richness (12 species) found 450m from the highway. *Pterostichus melanarius* was the most abundantly collected species on the grid, followed by *Scaphinotus marginatus* Fischer (85 individuals)and *Pterostichus adstrictus* (45 individuals). Activity density of *Pterostichus melanarius* was similar to the earlier data up to 200 m into the forest; however, there was a clear increase in activity density at each sampling location beyond 200 m (Figure 5a), suggesting that the species is still colonizing the deciduous forest at George Lake.

In contrast to data from the previous decade (Niemelä and Spence 1999), data from 2007-08 showed no significant correlation between distance from forest edge and %LW (Spearman r = 0.29, P > 0.05). In fact, %LW appears to have decreased in the forest interior (Figure 5b) compared to previous years. However, SW individuals clearly increased in the forest interior. The %SW captured >100m from the highway was significantly higher in 2007-08 than in 1991-92 (Χ^2^ = 4.56, P < 0.05). In contrast, %SW captured between the verge and 100 m into the forest interior did not change over the years (Χ^2^= 0.0039 , P > 0.05), suggesting that the wing-morph ratio has stabilized somewhat locally at about 44% LW, much higher than has been observed in Europe.

**Figure:**
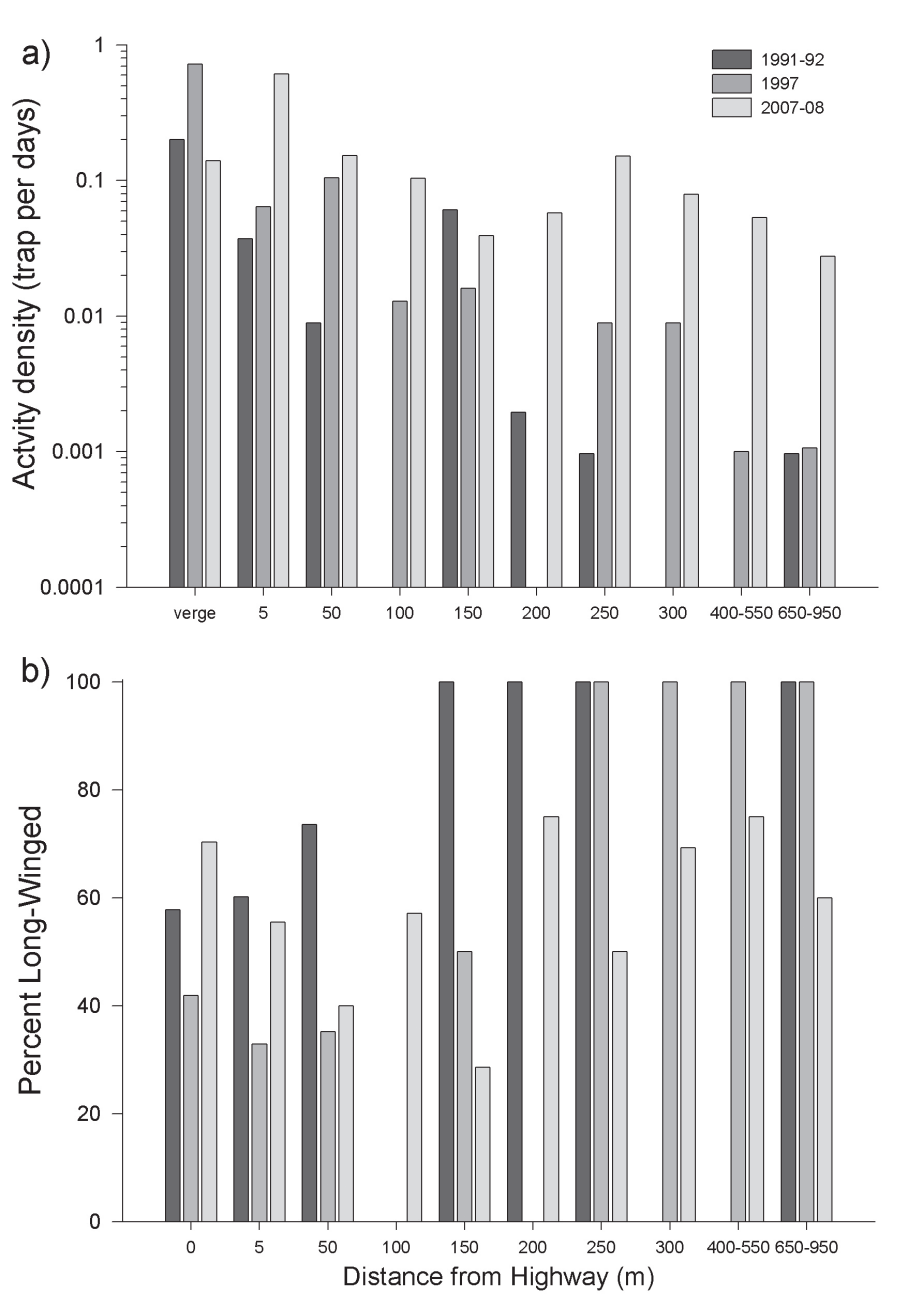
**Figure 5.** Distributions of *Pterostichus melanarius* pitfall trapped at George Lake, starting in the road verge and extending 950 m into the forest for three sampling periods: 1991-92, 1997 and 2007-08 (see text for details). (a) Activity density standardized by the number of traps and number of days traps were in operation. In 2007 and 2008, activity density was adjusted for shorter sampling period as explained in the text. (b) Percentage of *Pterostichus melanarius* that was macropterous. Captures from some transects are pooled for presentation.

## Discussion

### Large scale movement across an urban-rural gradient

We found a clear pattern of increasing frequency of LW *Pterostichus melanarius* with increasing distance from the centre of Edmonton, using data from 1998 and 1999, collected forty years after the first report of this species in the city. Because a similar pattern was not reflected for the native wing-dimorphic species *Agonum retractum*, we suggest that the pattern in *Pterostichus melanarius* did not result from general response to some underlying habitat gradient. Instead, we propose this is evidence that *Pterostichus melanarius* is spreading outward onto the rural landscape from a local centre of high population in Edmonton through flight of LW individuals, as suggested in Lindroth’s general hypothesis (Lindroth 1949) .

In 1998-99 populations of *Pterostichus melanarius* appeared to be more stable in the urban area but were still expanding in the rural area. Data collected in 2007, however, suggest that the %LW individuals in the rural area had dropped significantly. Because the frequency of the recessive LW trait (Aukema et al. 1996) was high in the rural population of *Pterostichus melanarius* in 1998-99, we suggest that these populations had been recently founded by LW individuals of both sexes that had flown in from sources closer to the city centre. The closest known sources would have been further to the west in the Edmonton area. As the colonization front passed, the dominant SW trait appears to be slowly replacing the LW trait at the rural end of our gradient. Replacement of the LW morph through local competitive advantage may be slowed in *Pterostichus melanarius* because continuing arrival of LW males continues to bring a large number of individuals carrying the LW locus into populations at the edge of an expanding range.

Our data are in agreement with the hypothesis that LW *Pterostichus melanarius* are responsible for movement associated with the early vanguard of first colonization and that over a relatively short time (in this case, about one decade), the SW morph becomes the dominant phenotype. Our data also support the hypothesis that population age can be roughly estimated using the frequency of LW in a population. After about 50 years, the frequencies of the SW phenotype in *Pterostichus melanarius* of urban and suburban areas have risen considerably, although they are still short of levels characteristic of established European populations, e.g., around 98% (den Boer and Van Dijk 1996).

Our study also reinforces the view that given sufficient time, introduced species like *Pterostichus melanarius* can become established in at least some natural habitats in North America. All the non-native species reported here and several others have previously been recorded far from urban areas in agricultural land and disturbed habitats (Bourassa et al. 2008; Butts et al. 2003; Cárcamo et al. 1995; Spence and Spence 1988). However, their steady incursion into aspen forest confirms that these invaders will not be restricted to anthropogenic habitats and that their integration into the North American fauna is well underway. There is little experimental evidence for short-term negative effects on the native fauna in natural habitats (Niemelä et al. 1997) however, the observed domination by introduced species of the ground beetle fauna in natural habitat reserves within the urban area suggests that long term effects could be significant and should be monitored.

It is not presently clear how to best interpret the high captures of non-native carabids in urban areas (Figure 3), where they accounted for 85.3 % of the total catch in this study. It is presently impossible to separate two possible explanations: 1) human disturbances in the city landscape favour these non-native, synanthropic species, either by direct habitat optimization effects that open up ‘niche space’ accessible mainly to the introduced species, or through broader landscape effects that increase the local pool of colonists every season, and 2) their high populations, even in urban aspen patches, simply reflect a longer history of these local populations. However, the lack of change in activity density of native carabids along the urban-rural gradient (see Figure 4) suggests that 1) urbanization around sufficiently large urban forest patches does not have a strong impact on the native fauna, and/or 2) the impact of introduced carabids on the native species is low. Defining the potential roles of inter-specific competition and intraguild predation in these systems awaits further research.

### Small scale dispersal at George lake

This study and previous work (Niemelä et al. 1997) clearly shows that the aspen forest at George Lake is a suitable habitat for the survival and reproduction of *Pterostichus melanarius* . In samples collected in the 1990s, the interior forest harboured mainly LW *Pterostichus melanarius*, exclusively so >250 m north of the Highway. In 2007-08, however, SW *Pterostichus melanarius* were trapped across the full N-S range of the grid, and there had been a clear decrease in the frequency LW among individuals deeper in the forest. We suggest that this pattern indicates the population in the interior forest descended from LW colonists and that this species has become resident, despite relatively small increases in activity density. It is possible that the beetles of the SW morph will be more competitive or more efficient reproducers, as has been suggested for other insects (Zera and Denno 1997) and that over time a dramatic increase in activity density will follow. Our data provide the baseline necessary to test this hypothesis through further observation.

In contrast to the high and relatively stable activity density of *Pterostichus melanarius* in the road verge, there was a general increase ofactivity density in the forest, especially at sites >200 m north of the highway. As suggested by Niemelä and Spence (1991, 1994), the present activity density might be too low to drive significant impacts on the native carabid fauna. However, if populations of *Pterostichus melanarius* continue to increase, these could have negative effects on the native carabid fauna through predation or intraguild competition (Currie et al. 1996). Nonetheless, the available data (e.g., Niemelä et al. 1997) suggest that introduced carabid species are occupying empty niches, rather than competitively excluding native carabids. Thus, this system provides provocative and interesting material for study of principles of community assembly.
